# Metastatic Gastric Tumors: Clinical and Endoscopic Features

**DOI:** 10.7759/cureus.58678

**Published:** 2024-04-21

**Authors:** Kazuki Hirano, Kosuke Nomura, Yorinari Ochiai, Junnosuke Hayasaka, Yugo Suzuki, Yutaka Mitsunaga, Hiroyuki Odagiri, Akira Masui, Daisuke Kikuchi, Shu Hoteya

**Affiliations:** 1 Gastroenterology, Toranomon Hospital, Tokyo, JPN

**Keywords:** melanoma, stomach cancer, esophageal cancer, breast cancer, endoscopy, gastric tumors, metastasis

## Abstract

Introduction: Stomach metastasis is rare, and there are few reports on its endoscopic features. Herein, we focused on the endoscopic features and discussed and reviewed the clinicopathological characteristics of metastatic gastric tumors.

Methods: We conducted an analysis on the clinicopathological features of individuals with gastric metastases originating from solid organ tumors at the Department of Gastroenterology, Toranomon Hospital, Minato-ku, Tokyo, Japan. Thirty-one cases were identified and evaluated for histology, initial presentation, endoscopic findings, lesion locations, treatment courses, and overall survival of the patients.

Results:Endoscopic findings resembling submucosal tumors were present in five cases (16%), and those with a morphology similar to that of primary gastric cancer were present in 26 cases (84%). In addition, seven patients (22%) were diagnosed with gastric metastasis due to a suspected biopsy of early gastric cancer. Solitary metastasis (21 patients, 67.7%) was more common than multiple metastases (10 patients, 32.2%). The median time from primary tumor to diagnosis was 36 months, and survival after metastasis was 19 months. The overall survival (OS) after the diagnosis of the primary tumor was 22 months for esophageal cancer, 25 months for lung cancer, and 100 months for breast cancer, and the OS after the diagnosis of gastric metastasis was almost the same. The average time from the diagnosis of the primary tumor to the diagnosis of gastric metastasis (*timespan) was more than seven years for breast and kidney cancers.

Conclusion: As the prognosis of patients with cancer gradually improves, they develop metastases more frequently. Understanding the endoscopic findings and information about a patient’s clinical history is useful to correctly diagnose gastric metastases.

## Introduction

Metastasis to the stomach is rare, reported to be 0.2-1.7% [1,2]. Regarding the macroscopic appearance of metastatic gastric tumors, multiple lesions and a bull’s eye sign on radiographs have been described as characteristic findings [3,4]. However, occasional case reports have described various endoscopic appearances without using conventional characteristics [5]. ﻿Although metastatic gastric tumors may originate from various organs, primary tumors that most commonly metastasize to the stomach include melanoma, breast, lung, and esophageal carcinomas [5,6]. As metastatic tumors in the stomach are rare, limited information is available regarding gastric metastases. Consequently, gastric metastases are often not considered in clinical practice [5].

This study reviewed the clinicopathological indices of a large number of patients with gastric metastases from malignant solid tumors.

## Materials and methods

Patients

We conducted a chart review of patients with endoscopically detected metastatic tumors in the stomach between April 2012 and December 2022 at the Department of Gastroenterology, Toranomon Hospital, Minato-ku, Tokyo, Japan. ﻿ ﻿Patients with no endoscopic findings in the medical record, no histological confirmation, leukemia, malignant lymphoma, or direct invasion from adjacent organs were excluded from the analysis. Finally, 31 patients with metastatic gastric tumors were included, and their medical records were retrospectively reviewed. The Ethics Committee of Toranomon Hospital provided approval for this study (No. 2539).

Endoscopic patterns of metastatic gastric lesions

**Figure 1:**
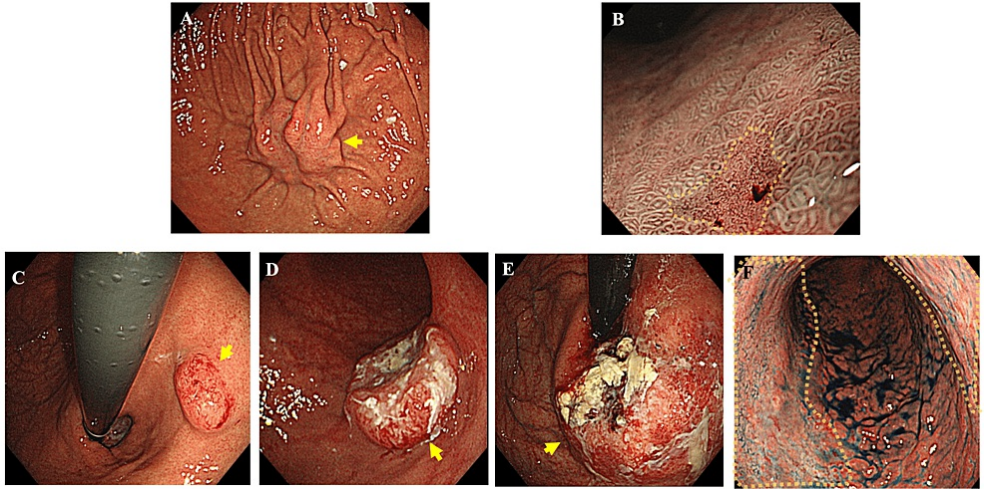
Endoscopic appearance of metastatic gastric tumors. (A) Resembling submucosal tumors caused by pancreatic cancer. Yellow arrows indicate tumors. (B) Resembling early primary gastric cancer caused by breast cancer. The area circled by the yellow dotted line indicates the tumor. (C–F) Resembling advanced gastric cancer: (C) type 1, esophageal squamous cell carcinoma (yellow arrows indicate tumors); (D) type 2, esophageal squamous cell carcinoma (yellow arrows indicate tumors); (E) type 3, esophageal squamous cell carcinoma (yellow arrows indicate tumors); (F) type 4, breast cancer (the area circled by the yellow dotted line indicates the tumor).

Esophagogastroduodenoscopy (EGD) was used for diagnosis, and all lesions were identified by endoscopic biopsy, followed by histologic analysis. ﻿ Data collected included epidemiologic characteristics, symptoms, indications for endoscopy, macroscopic symptoms, time from diagnosis of primary tumor to detection of gastric metastases, and treatment. Endoscopy was performed using a single-channel endoscope(GIF-H260, H290, and H290Z; Olympus Optical Co., Ltd., Tokyo, Japan). Using standard biopsy forceps and direct vision endoscopy, biopsies were obtained from suspected lesions. ﻿Endoscopic gross findings were classified into three main patterns: resembling submucosal tumors, resembling primary gastric cancer, and resembling advanced cancer. Those resembling advanced cancer were classified according to Bormann's classification (Figure 1).

Histological patterns of metastatic gastric lesions

All histological results were reviewed by pathologists. Biopsy specimens were reviewed in detail and compared to the histologic characteristics of the primary tumor.

Statistical analysis

Categorical variables underwent analysis employing Fisher’s exact test, while the Mann-Whitney U test was utilized for continuous variables. Continuous variables are depicted as medians with interquartile ranges (IQR), while categorical variables are represented as counts and percentages. The Kaplan-Meier method and log-rank test were used to evaluate differences in overall survival. All p-values were two-sided, and p < 0.05 was considered statistically significant. All statistical analyses were performed using ﻿EZR version 1.42 (Saitama Medical Center, Jichi Medical University, Saitama, Japan) [7].

## Results

Baseline characteristics of patients with metastatic gastric tumors

﻿The baseline characteristics of the patients are summarized in Table 1. The study population consisted of 18 men and 13 women (median age, 67.3 years; IQR, 63.5-71.0 years).

**Table 1:** Sites of the primary tumors (N = 31)

Primary tumor (N = 31)	n (%)
Esophagus	13 (42)
Breast	8 (26)
Lung	3 (10)
Malignant melanoma	3 (10)
Kidney	2 (6)
Pancreas	1 (3)
Angiosarcoma	1 (3)

The main clinical manifestations included ﻿dysphagia (five patients, 16.1%), gastrointestinal bleeding (five patients, 16.1%), abdominal pain (four patients, 12.9%), and vomiting (two patients, 6.4%). Meanwhile, 15 patients had no symptoms; among these, metastatic gastric tumors were detected in five (16.1%) by routine computed tomography or positron emission tomography, five (16.1%) by preoperative examination, two (6.4%) who were referred to a previous hospital due to suspicion of early-stage gastric cancer, two (6.4%) by endoscopy for chronic gastritis follow-up, and one (3.2%) by endoscopy for follow-up after esophageal cancer surgery.

Endoscopic findings revealed that solitary metastases (21 patients, 67.7%) were more common than multiple metastases (10 patients, 32.2%). The median interval between the diagnosis of the primary tumor and that of the metastatic gastric tumor was 13.5 months (IQR, 0.75-33.5 months).

Sites of primary tumors

As shown in Table 2, the most common primary malignancy associated with gastric metastasis was esophageal carcinoma (13 cases, 42%), followed by breast cancer (eight cases, 26%), lung cancer (three cases, 10%), malignant melanoma (three cases, 10%), kidney cancer (two cases, 6%), pancreatic cancer (one case, 3%), and angiosarcoma (one case, 3%). Among the 13 cases of esophageal cancer, 11 were squamous cell carcinomas, one was an adenocarcinoma, and one was a neuroendocrine carcinoma.

**Table 2:** Baseline characteristics of patients with metastatic tumors with the stomach(N=31) Data are presented as number of patients (%) or median (interquartile range). The data for age and sex are as follows: age = 67.3 (63.5-71.0), sex ratio = male:female = 18:13.

Clinical feature (N = 31)	Value
Main symptom (%)	n (%)
Symptoms present	16 (51.6)
Epigastric pain	4 (12.9)
Dysphagia	5 (16.1)
GI bleeding	5 (16.1)
Vomiting	2 (6.4)
No symptoms	15 (48.4)
Detected by CT or MRI	5 (16.1)
Detected by preoperative endoscopy	5 (16.1)
Referred from previous hospital	2 (6.4)
Endoscopy for Chronic Gastritis Follow-up	2 (6.4)
Endoscopy for follow-up after esophageal cancer surgery	1 (3.2)

Endoscopic characteristics of metastatic gastric tumors

﻿Table 3 shows the endoscopic characteristics of metastatic gastric tumors, which were used to divide the patients into two groups (resembling submucosal tumors (five patients, 16%) and resembling primary gastric cancer (26 patients, 84%)). Among patients with metastases that resembled early gastric cancer, four, four, two, two, and one had esophageal cancer, breast cancer, malignant melanoma, kidney cancer, and angiosarcoma, respectively. Among patients with metastases that resembled advanced gastric cancer, eight, two, one, and one had esophageal cancer, breast cancer, lung cancer, and malignant melanoma, respectively﻿. Although gastric metastases may be recognized as abnormalities on EGD, no characteristic features can be used to identify them because of the variable morphology of the tumors.

**Table 3:** Summary of characteristics of the 31 patients Data are duplicated due to the presence of multiple cases. SMT, resembling submucosal tumor. EGC, resembled early gastric cancer. AGC, resembling advanced gastric cancer. U, upper third of the stomach. M, middle third of the stomach. L, lower third of the stomach.

		All (N = 31)	Esophageal cancer (N = 13）	Breast cancer (N = 8)	Lung cancer (N = 3)	Malignant melanoma (N = 3)	Kidney cancer (N = 2)	Others (N = 2)
Gross appearance	SMT	5	1	2	1	0	0	1
	EGC	14	4	4	1	2	2	1
	AGC	12	8	2	1	1	0	0
Tumor location	U	22	12	5	0	2	1	2
	M	12	1	4	3	1	1	2
	L	7	0	4	0	2	0	1
Tumor circumference	Lesser curvature	14	11	1	0	0	1	1
	Greater curvature	11	1	4	2	2	1	1
	Anterior wall	5	1	2	1	1	0	0
	Posterior wall	5	1	2	0	0	0	2
No. of lesions	Solitary	21	13	2	3	1	2	0
	Multiple	9	0	5	0	2	0	2

**Figure 2:**
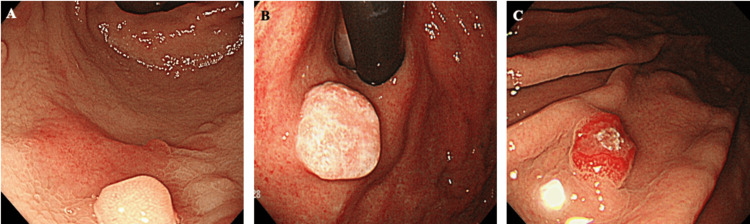
Case of suspected gastric metastasis by endoscopy (A) Breast cancer. (B) Esophageal cancer. (C) Kidney cancer: clear cell carcinoma.

It is important to note that seven patients (22%) were diagnosed with gastric metastasis through biopsy for suspected early gastric cancer (Table 4, Figure 2). 

**Table 4:** Gastric metastasis as a result of a suspected early gastric cancer biopsy (N = 31)

	All (N = 31)	Esophageal carcinoma (N = 13）	Breast cancer (N = 8)	Lung cancer (N = 3)	Malignant melanoma (N = 3)	Kidney cancer (N = 2)	Others (N = 2)
Cases (No.)	7	1	4	1	0	1	0

Location

﻿Solitary metastases (21 patients, 67.7%) were more common than multiple metastases (10 patients, 32.2%). Solitary lesions were mainly located in the upper third (71%) of the stomach. Of the patients with solitary lesions, 13 had esophageal cancer that metastasized to the lesser curvature. In addition, most cases of breast cancers had multiple gastric metastases (six cases, 75%) to the greater curvature. 

﻿Clinical outcomes of metastatic gastric tumors according to treatment

﻿The clinical data of the 31 patients are summarized in Table 5. Of the 30 patients who received treatment for the primary lesion, five were converted to best supportive care (BSC) after the diagnosis of gastric metastasis. Among patients with esophageal cancer who had both a primary tumor and gastric metastasis, surgery was performed after the diagnosis of metastasis in three patients.

**Table 5:** Clinical outcomes of metastatic tumors

	All (N = 31)	Esophagus (N = 13)	Breast (N = 8)	Lung (N = 3)	Others (N = 7)
Treatment method of primary cancer					
Surgery (+chemotherapy and/or radiotherapy)	12	4	5	1	2
Chemotherapy and/or radiotherapy	18	8	3	2	5
Conservative	1	1	0	0	0
Treatment method for metastatic cancer					
Surgery (+chemotherapy and/or radiotherapy)	4	3	0	0	1
Chemotherapy and/or radiotherapy	21	9	5	2	5
Conservative therapy	6	1	3	1	1

**Figure 3:**
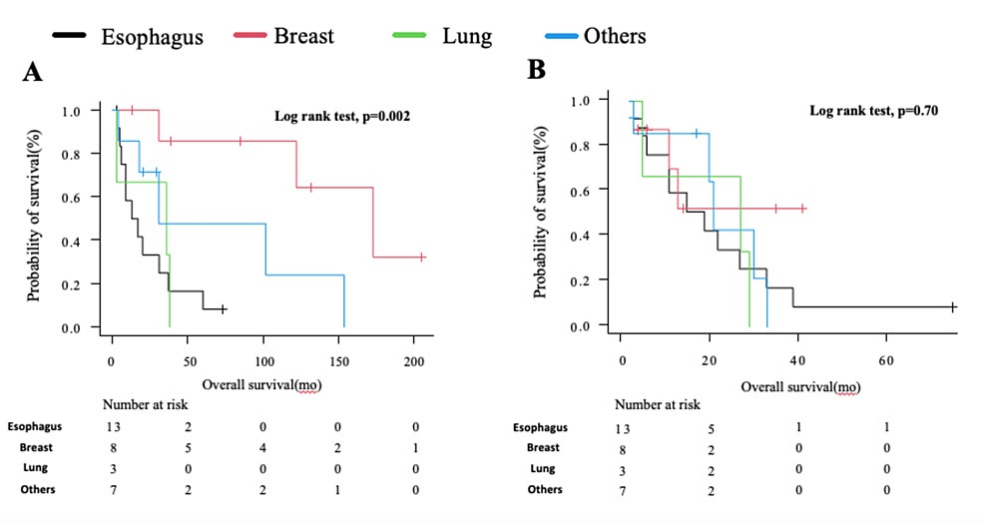
Kaplan-Meier survival curve showing survival from the diagnosis of the primary tumor (A) Overall survival from the diagnosis of the primary tumor and (B) from the diagnosis of gastric metastasis.

One case of postoperative recurrence of renal cancer was treated with surgical resection. The median time from the primary tumor to diagnosis of gastric metastasis was 36 months, and survival after metastasis was 19 months. The OS from diagnosis of the primary tumor was 22 months for esophageal cancer, 25 months for lung cancer, and 100 months for breast cancer. The OS from the time of diagnosis of gastric metastasis was similar (Figure 3A, 3B).

**Figure 4:**
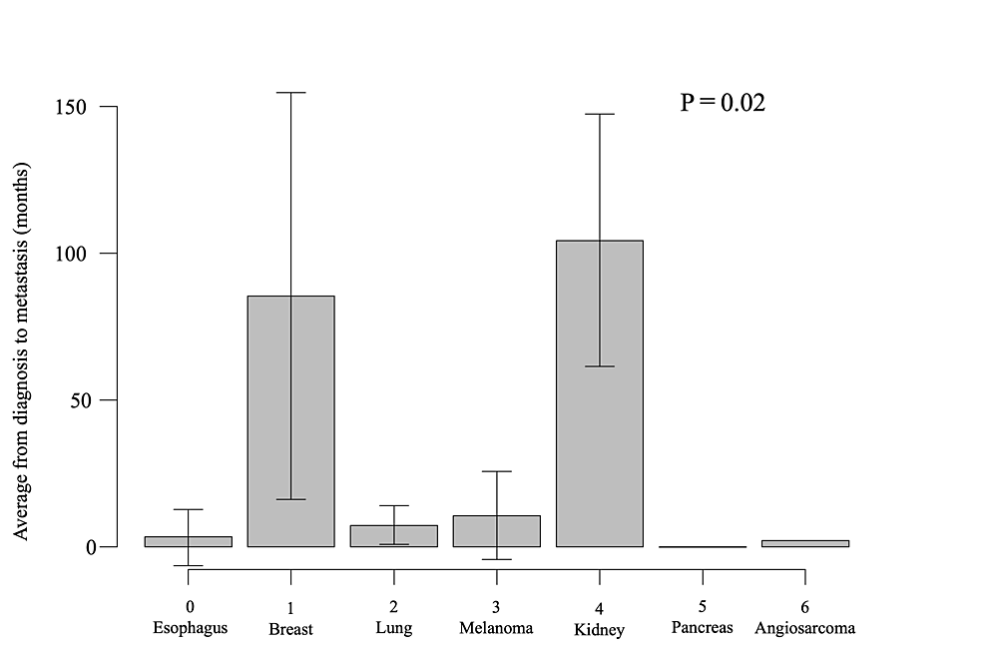
Timespan* in each carcinoma Bar graph showing the *timespan and standard deviation for each carcinoma (P = 0.02). *Time between the diagnosis of the primary tumor and metastasis.

The time from the diagnosis (*timespan) of the primary tumor to the diagnosis of gastric metastasis, breast cancer, and kidney cancer was more than seven years on average (breast cancer: 85 months, kidney cancer: 105 months, P = 0.02, Figure 4).

## Discussion

The stomach rarely develops metastasis. Hence, it is difficult to assess the actual incidence of gastric metastasis. Only 0.2-1.7% of patients with metastatic cancer develop metastasis to the stomach, and the diagnosis is usually made on autopsy [1,2].

However, as the prognosis of patients with cancer has gradually improved, gastric metastases have become more frequent [5]. In this study, we discussed the clinical characteristics of metastatic gastric tumors at our institution by focusing on endoscopic findings and comparing them with those in previous reports.

Previous studies have shown that endoscopic patterns, such as multiple nodules, bull’s eye patterns, exogenous mass lesions, ulcerations, and polypoid tumors, are common and that characteristic endoscopic findings, such as doughnut-shaped raised lesions and volcanic ulcers, are useful for the diagnosis of gastric metastasis [5]. However, some cases of gastric metastasis are difficult to differentiate from primary gastric cancer [8,9], rendering it even more challenging to assume that the gastric tumor is primary; therefore, it is important to accumulate additional cases. In this study, more than half of the cases were solitary metastases, and although they varied with respect to morphology, about half showed early gastric cancer-like morphology; in fact, seven cases (22%) were suspected to be early gastric cancer and were biopsied. Although there were no significant differences in the factors contributing to this finding, there were some differences in the primary sites that were more likely to develop gastric metastasis.

In this study, breast cancer tended to present with a gastric cancer-like morphology (four cases, 50%), even at an early stage, which occurred more frequently in the greater curvature. In some cases, treatment for gastric cancer was planned, whereas in others, gastric metastasis was diagnosed as the primary malignancy. This pattern suggests that hematogenous metastasis may be the underlying cause. By contrast, most esophageal carcinomas had an advanced gastric cancer-like morphology (eight cases, 62%), were solitary (13 cases, 100%), and developed on the lesser curvature of the upper gastric body. This finding suggests that lymphatic metastasis may be the underlying cause. Further elucidating the nuanced characteristics of these metastatic patterns is critical for optimizing diagnostic and therapeutic approaches. For instance, understanding the propensity of breast cancer to mimic gastric cancer morphologically, especially along the greater curvature, can inform clinicians to consider metastatic disease in the differential diagnosis, potentially avoiding misdiagnosis and facilitating prompt intervention. Similarly, the distinct distribution and morphology of esophageal carcinoma metastases, primarily situated on the lesser curvature of the upper gastric body, underscore the importance of recognizing the lymphatic route as a predominant pathway for dissemination in this context. By delineating these distinctive metastatic patterns, clinicians can refine their clinical acumen and tailor management strategies accordingly, ultimately enhancing patient care and prognostic outcomes.

Previous studies have reported that after the diagnosis of metastatic cancer, patients with a solitary disease have longer survival than those with multiple lesions [3]. In this study, no statistically significant differences were observed. This is probably due to the fact that most of the cases of solitary lesions involved primary esophageal cancer in this study.

Most patients with metastatic gastric tumors are asymptomatic, and symptoms of metastatic tumors, such as pain, nausea, vomiting, and bleeding, are nonspecific [6]. In a previous autopsy series, abdominal pain was the most common symptom, followed by nausea, vomiting, anorexia, and acute upper gastrointestinal bleeding [6]. Of the 31 cases in this study, gastrointestinal bleeding, iron-deficiency anemia (five cases, 16.1%), and dysphagia (five cases, 16.1%) were the most common presentations, indicating that endoscopy is an important diagnostic tool for evaluating outcomes of therapeutic interventions.

We believe that endoscopic and histological evaluation should be performed to identify metastatic gastrointestinal lesions in patients with known primary cancer presenting with gastrointestinal symptoms, especially abdominal pain and bleeding [9-12].

Previous studies have shown that the mode of gastric metastasis is not clearly understood and may vary by primary site, with the breast and lungs being the most common primary sites [6], which may reflect the high incidence of these tumors in the general population.

There have also been many reports of malignant melanoma possibly because of the higher incidence of gastric metastases from primary malignant melanoma owing to the higher number of tumors directed to the gastrointestinal tract [6].

According to the literature, about half of patients with gastric metastases also have metastatic lesions in other organs, and the average time from diagnosis of gastric metastasis to death is about 4.75 months [13]. In the present study, 28 patients (90%) had metastatic disease in other organs, including lymph nodes, and the time from diagnosis of gastric metastasis to the end of the observation period was approximately 16 months.

The treatment of gastric metastases is shown in Table 5; however, metastases were resected in only three cases of esophageal cancer and metastases of renal cancer. A previous report stated that resection of gastric metastases of renal cancer is also useful to control bleeding [14], and resection of gastric metastases to control bleeding may be useful for patients with a relatively good prognosis after systemic treatment, such as those with breast and renal cancers [14,15].

The most common primary lesion in this study was esophageal cancer, which was found in 13 patients, more than those in previous reports. Two patients had liver or bone metastases other than gastric metastasis and died within approximately two months, whereas others only had lymph node metastasis and could be treated with chemotherapy or radiation therapy, resulting in a mean survival of approximately 21 months (Table 5). 　 

We believe that endoscopy should be performed with caution and that gastric metastases should be considered not only when symptoms are evident but also in the absence of symptoms, especially in those with esophageal and breast cancer. Symptomatic symptoms, especially in non-gastrointestinal cancers undergoing treatment, may be considered a side effect of chemotherapy and may delay detection [9,16-18]. Moreover, the study highlighted a significant disparity in the timespan from the diagnosis of the primary tumor to the discovery of gastric metastasis (*timespan). For breast cancer and renal cancer, this duration exceeded seven years on average, emphasizing the need for continued vigilance even years after the initial cancer diagnosis. By contrast, the interval was notably shorter for other carcinomas, reflecting differences in disease behavior and surveillance requirements. This discrepancy underscores the necessity for prolonged and meticulous monitoring in certain cancer types, particularly breast and renal cancers, where metastases may manifest after prolonged latency periods (Figure 4). Despite its valuable insights, this study is constrained by its retrospective design and small sample size, alongside the inherent heterogeneity in primary malignancies and treatment modalities. Therefore, while it offers valuable insights, caution must be exercised in extrapolating its findings universally.

## Conclusions

This study sheds light on the increased incidence of gastric metastases and the complexity of diagnosis, especially in light of improving cancer prognosis. Endoscopic findings often resemble primary gastric cancer but exhibit subtle patterns reflecting the primary tumor site. This study highlights differences in metastatic patterns by primary tumor site, with breast cancer often mimicking the morphology of gastric cancer and esophageal cancer showing distinct distribution and morphology. The reason was thought to be that esophageal carcinoma is often a solitary lesion, but no significant difference was found in this study. Solitary lesions generally have a better survival prognosis, whereas symptomatic lesions are nonspecific and require caution in diagnosis. Treatment strategies vary, and surgical resection is considered in select cases. Long-term follow-up is especially important for breast and renal cancers, as metastases may appear many years after initial diagnosis. However, the retrospective design of this study and the small sample size emphasize the need for further research.

## References

[1] Menuck LS, Amberg JR (1975). Metastatic disease involving the stomach. Am J Dig Dis.

[2] Kadakia SC, Parker A, Canales L (1992). Metastatic tumors to the upper gastrointestinal tract: endoscopic experience. Am J Gastroenterol.

[3] Scobie BA (1966). Malignant gastric ulcer due to metastasis. Australas Radiol.

[4] Richter R, Panish J, Berci G (1972). Endoscopic findings in melanoma metastatic to the stomach. Gastrointest Endosc.

[5] Namikawa T, Hanazaki K (2014). Clinicopathological features and treatment outcomes of metastatic tumors in the stomach. Surg Today.

[6] Kim GH, Ahn JY, Jung HY (2015). Clinical and endoscopic features of metastatic tumors in the stomach. Gut Liver.

[7] Kanda Y (2013). Investigation of the freely available easy-to-use software 'EZR' for medical statistics. Bone Marrow Transplant.

[8] Hsu CC, Chen JJ, Changchien CS (1996). Endoscopic features of metastatic tumors in the upper gastrointestinal tract. Endoscopy.

[9] Jones GE, Strauss DC, Forshaw MJ, Deere H, Mahedeva U, Mason RC (2007). Breast cancer metastasis to the stomach may mimic primary gastric cancer: report of two cases and review of literature. World J Surg Oncol.

[10] Kim YI, Kang BC, Sung SH (2013). Surgically resected gastric metastasis of pulmonary squamous cell carcinoma. World J Gastrointest Surg.

[11] Reiman T, Butts CA (2001). Upper gastrointestinal bleeding as a metastatic manifestation of breast cancer: a case report and review of the literature. Can J Gastroenterol.

[12] Oda Oda, Kondo H, Yamao T (2001). Metastatic tumors to the stomach: analysis of 54 patients diagnosed at endoscopy and 347 autopsy cases. Endoscopy.

[13] Campoli PM, Ejima FH, Cardoso DM (2006). Metastatic cancer to the stomach. Gastric Cancer.

[14] Namikawa T, Munekage M, Kitagawa H, Okabayashi T, Kobayashi M, Hanazaki K (2012). Metastatic gastric tumors arising from renal cell carcinoma: clinical characteristics and outcomes of this uncommon disease. Oncol Lett.

[15] Sakurai K, Muguruma K, Yamazoe S (2014). Gastric metastasis from renal cell carcinoma with gastrointestinal bleeding: a case report and review of the literature. Int Surg.

[16] Taal BG, Peterse H, Boot H (2000). Clinical presentation, endoscopic features, and treatment of gastric metastases from breast carcinoma. Cancer.

[17] Suzaki N, Hiraki A, Ueoka H (2002). Gastric perforation due to metastasis from adenocarcinoma of the lung. Anticancer Res.

[18] Kim MJ, Hong JH, Park ES, Byun JH (2015). Gastric metastasis from primary lung adenocarcinoma mimicking primary gastric cancer. World J Gastrointest Oncol.

